# Benign adenomyoepitelioma of the breast: Presentation of two rare cases and review of literature

**DOI:** 10.1016/j.ijscr.2020.01.010

**Published:** 2020-01-23

**Authors:** Eva Intagliata, Santi Gangi, Claudio Trovato, Rosario Vecchio, Angela Strazzanti

**Affiliations:** Department of General Surgery and Medical Surgical Specialties, University of Catania, Policlinico Vittorio Emanuele Hospital, Via S. Sofia 78, 95123 Catania, Italy

**Keywords:** Breast adenomyoepithelioma, Adenomyoepithelial tumors, Rare breast tumors

## Abstract

**Introduction:**

Adenomyoepithelioma of the breast is a rare benign breast neoplasm with a particular behaviour.

**Presentation of cases:**

We report two cases of adenomyoepithelioma of the breast in two old female patients, diagnosed over a period of 5 years. The clinical presentation, the radiological and the histopathologic findings are discussed.

**Discussion:**

Adenomyoepithelioma of the breast is characterized by the proliferation of both epithelial and myoepithelial cells belonging to the breast lobules and ducts. The imaging features are not pathognomonic and FNAB is usually not diagnostic. The morphologic appearance of this tumor varies, and it has to be considered in the differential diagnosis with other breast tumors. Although benign, adenomyoepithelioma has a potential for local recurrence, and malignant transformation is possible; therefore, wide excision is recommended to lower the recurrence rate.

**Conclusion:**

The rarity of this histological type and the finding of two cases in a relatively short period makes this case report unique.

## Highlights

• Adenomyoepithelioma of the breast is a rare benign breast neoplasm.
• The rarity of this histological type of benign breast tumor and the finding of two cases in a short period makes this case report unique.
• Although benign, adenomyoepithelioma has a potential for local recurrence; therefore, wide excision is recommended.

## 1 Introduction

Adenomyoepithelioma is a rare benign breast neoplasm characterized by the proliferation of both epithelial and myoepithelial cells belonging to the breast lobules and ducts.

Adenomyoepithelial tumors may be found in salivary glands and skin appendages, whereas its finding in the mammary gland is very rare [1].

In the few cases reported in the Literature, these tumors clinically show up as a single asymptomatic hard roundish shape irregular nodule and the differential diagnosis with breast cancer is challenging [[2], [3], [4], [5], [6], [7]]. The radiologic and histologic appearance of this tumor varies, and it has to be considered in the differential diagnosis with other breast tumors.

Although benign, adenomyoepithelioma has a potential for local recurrence; therefore, proper excision with margins free from disease is mandatory for diagnosis and treatment. Malignant transformation is possible under certain conditions, and in this case, the tumor must be treated as a breast carcinoma [[8], [9], [10]].

We report two cases of adenomyoepithelioma of the breast in two old female patients, diagnosed over a period of 5 years in our Institute.

The work has been reported in line with the SCARE criteria [11].

## 2 Cases reports

### 2.1 Case 1

A 63-year-old female presented with a right breast lump that was described as fluctuating in size over the month. She denied other changes in her breasts including nipple inversion or discharge, erythema. She had a normal mammogram one year prior. A mammogram was performed one month later showed a well-defined lobulated opacity in nodule in a hard right-angled, roundish shape of the right breast. Ultrasound confirmed a 20 × 15 × 15 mm complex cyst region of the right breast. In the right axilla, a prominent lymph node measuring 11 × 10 × 10 mm was visualized without malignant aspects. She had FNAc. The cytological examination showed typical epithelial hyperplasia, and modulated cellularity. As the diagnosis was not relevant, the patient was subjected to lumpectomy. The lumpectomy specimen revealed well-circumscribed, firm, globular mass with gray-white, solid appearance on cut section. The definitive histological examination was breast adenomyopithelioma with intraductal epithelial hyperplasia without atypia. S 100, Actina ML, p63, EMA (epithelial membrane antigen), Ck (cytokeratin), AE1-AE3 positive. Although myoepithelial cells were predominant, there were numerous benign epithelial cells too, including bare nuclei and occasional fibrous stromal elements.

### 2.2 Case 2

A 72 years old female presents to our observation with a diagnosis of fibroadenoma of the inner inferior quadrant of the left breast performed in another Institute. The lesion presented to our observation as an asymptomatic nodule in a hard right-angled, roundish shape with a size of 2 cm. Mammography showed a 15 × 13 × 15 mm nodule with clear and defined margins, depositing by benign pathology, the ultrasound showed a similar corpuscular nodule. She refused breast MRI and FNAc, therefore she underwent lumpectomy. Frozen sections showed an encapsulated and highly cellular lesion consisting of tubules. The tubules were lined by an attenuated lining of ductal epithelial cells with bland nuclei and small nucleoli, surrounded by a prominent layer of clear cells. No atypical mitosis or necrosis was seen. Final examination showed adenomyoepithelioma of the breast. Immunohistochemistry revealed epithelial cells positive for CK and EMA, while the myoepithelial cells were clearly demonstrated by positivity with calponin and p63. Surgical margins were disease-free.

## 3 Discussion

Breast adenomyoepithelioma was first reported by Hamperl in 1970 [12].

Adenomyoepithelial tumors of the breast are extremely rare neoplasms; approximately 150 cases have been described in the Literature [13]. They have been described in 26–81 years old patients, and the incidence increases with age [14].

Adenomyoepithelial tumors of the breast form a group of rare tumors having a variable behavior [15]. Adenomyoepithelial tumors are characterized by proliferation of both epithelial and myoepithelial cells, which belong to the breast lobules and ducts [16]. Rarely, male breast can be affected [17].

According to the WHO, the benign variant is called adenomyoepithelioma, while the malignant variant must be defined by the histology of the malignant component [18].

Benign adenomyoepithelioma has been classified as tubular (ill-defined margin like tubular adenoma), lobulated (nests of myoepithelial cells surrounding compressed epithelial lined spaces) or spindle cell (sparse epithelial lined spaces leiomyoma-like) variants based on their growth patterns [13].

Malignant transformation may occur in tumors >1,6 cm [10], with high mitotic rate, atypia, necrosis, cellular pleiomorphism and infiltrative borders. Malignant changes may involve most often the epithelial cells than the myoepithelial ones, and rarely them both [19]. Malignant adenomyoepithelial tumors can be associated to mostly hematogenous rather than lymphatic metastasis [16] to lung [20], brain, jaws, liver and thyroid [14].

Histopathology of breast adenomyoepithelioma reveals as a well circumscribed encapsulated mass or as multinodular masses with proliferation of epithelial and myoepithelial cells. Aggregated lobules of glands with tall lining epithelium with scant eosinophilic cytoplasm and hypercromatic nuclei surrounded by myoepithelial cells with clear cytoplasm. Epithelial cells usually form glandular spaces. Myoepithelial cells may be prevalent and may be spindle shaped, clear or polygonal. Apocrine metaplasia or adenomyoepitheliomatous hyperplasia may be present. Moderate to high cellular with large clusters of epithelium and myoepithelium are present, and tubular structures can be occasionally found. Myoepithelium appears as small clusters or dispersed cells with epithelioid morphology, intranuclear or intracytoplasmatic vacuoles, and often-naked bipolar nuclei. Mild to moderate nucler atypia is present, while metachromatic fibrillary stroma can be occasionally found. Mitotic figures and necrosis are not present. Histological examination shows tubular or lobular structures with epithelial and myoepithelial components. Immunohistochemistry defines the epithelial component when markers cytocheratin AE1/AE3, CK7, CK8, EMA and CEA are found; the myoepithelial component is defined by markers S100, SMA, SMMCH, p63, CK5/14, smooth muscle myosin heavy chain [4,5,21]. There could be positivity for estrogen and/or progesterone receptors [22].

At electron microscopy, myoepithelial features include myofibrils with dense bodies, pinocytotic vesicles, desmosomes or tight junctions, patchy basement membrane.

Tubular variant and some lobular variants with high mitotic rate >3 MF/10 HPF are associated with high incidence of recurrence [13].

Adenomyoepithelioma usually presents as a single palpable well circumscribed firm mass (mean 1–2 cm), whose dimension can be up to 8 cm. Rarely, satellite nodules or multiple breast masses may be found [15]. It usually occurs in the central portion of the breast and very rarely may be bilateral, with one case of bilateral onset reported in the Literature [13,15].

Differential diagnosis has to be set towards fibroadenoma suspicious for malignancy, adenosis tumor, intraductal papilloma, invasive carcinoma, nipple adenoma and tubular adenoma.

Radiologic and ultrasonographic findings are not specific and univocal and do not allow to discern the benign or malignant nature of the lesion [23]. MRI provides additional but not diriment information about morphological and hemodynamic features [24].

Most mammographic findings reported in the Literature describe the detection of oval or round isodense mass with clear or shaded or microlobulated margins in the context of a heterogeneous or an extremely dense parenchymal pattern. The superimposition of a very dense breast tissue might not allow the visualization of the tumor. Microcalcifications might sometimes be present inside the mass [14,16,24,25].

Common ultrasonographic findings include a solid hypoechoic oval [16], irregular nodule with irregular, microlobulated, or indistinct margins. Sometimes a complex echoic eco-texture with adjacent ductectasia, posterior acoustic enhancement and echogenic halo can be found. Color Doppler ultrasound shows increased mostly peripheral than central vascularity or no vascularity [14,25,26].

At MRI, Zhang et al. [24] found masses which clearly enhanced homogeneously with dynamic progressive enhancement curve in 3 patients affected by benign adenomyoepithelioma.

Fine needle aspiration was never diagnostic, as reported in the Literature and in our case [3,5,16]. These lesions can be diagnostically challenging, also when a core needle biopsy is performed, because of the heterogeneity of adenomyoepitheliomas [6].

In the Literature, malignant case are more often described, whereas benign adenomyoepithelioma are rarely reported.

Reis-Filho et al. [27] reported a case of a 48 year old woman with collagenous spherulosis in a benign adenomyoepithelioma of the breast presenting as a retroareolar, non-palpable nodule.

Hikino et al. [3] reported a case of a 56-year-old woman with a round, non-tender, not fixed mass measuring 30 mm in her left breast without palpable lymph nodes. Preoperative examination with mammography, sonography, computed tomography, and magnetic resonance imaging showed an intracystic tumor with an indistinct margin. Since the results of fine-needle aspiration cytology of the tumor was interpreted as carcinoma, partial mastectomy with dissection of the axillary nodes was performed. Intracystic benign adenomyoepithelioma of the breast was the final histologic diagnosis [3].

Zarbo et al. [28] described a case of cellular benign adenomyoepithelioma of the breast.

Howlett et al. [16] reported 3 female patients, whose age ranged from 69 to 74 years, with benign breast adenomyoepithelioma, who presented over a 1-year period with unilateral breast lesions without evidence of associated lymphadenopathy.

Currently, there are neither guidelines for the treatment of either benign or malignant adenomyoepithelioma nor mature clinical experience of reference, perhaps since the pathology is rare [20].

Yuan et al. [7] reported two cases of malignant breast adenomyoepitheliomas. The first patient has a 7 cm-nodule fixed and ulcerated; she did not undergo surgery and received neo-adjuvant radiochemotherapy, but the tumor did not shrink. The patient died from the recurrence at the chest wall and bone metastases 10 months after the radical resection. For the second patient affected by malignant adenomyoepithelioma combined with infiltrating ductal carcinoma measuring about 2 cm each, the adjuvant chemotherapy was carried out; because the hormone receptor of infiltrating ductal carcinoma was positive, the endocrinotherapy was conducted and the general condition of the patient was satisfactory at the follow-up.

Given the unclear and unpredictable propensity for malignant transformation and the risk of local recurrence, conservative excision with negative margins currently seems to be the appropriate surgical treatment [2,5,15,29]. In case of malignant adenomyoepithelioma, mastectomy and analysis of sentinel lymph node are recommended [[7], [8], [9], [10]].

Prognosis is good for benign adenomyoepithelioma and radiotherapy has been used in few cases with local recurrence [15].

Prognosis is poor in malignant cases since, the disease is characterized by low grade [30], invasiveness, high recurrence rate and metastasis [7], and chemotherapy has no much success [15].

## 4 Conclusions

In conclusion, adenomyoepithelioma is an unusual mostly benign breast neoplasm and should be considered in the differential diagnosis with other solid lesions of the breast. The imaging features are not pathognomonic, but the benign or malignant nature of the lesion might be suspected at X-ray or ultrasound examination. FNAB is usually not diagnostic. Currently, there are no guidelines for the medical and surgical treatment of both benign and malignant adenomyoepitheliomas. Surgical excision aiming at oncological radicality is the current therapeutic orientation, because of high recurrence rate for benign histological type and aggressiveness for the malignant one [31].

## Funding

There are no sources of funding.

## Ethical approval

The study is exempt from ethical approval in our Hospital in Italy.

## Consent

Written informed consent was obtained from the patients for publication of this case report and accompanying images. A copy of the written consent is available for review by the Editor-in-Chief of this journal on request.

## Author contribution

All the Authors (Intagliata Eva, Trovato Claudio, Gangi Santi, Vecchio Rosario, Strazzanti Angela) contributed to conceptualization, data curation, investigation, methodology and writing.

Intagliata Eva and Strazzanti Angela, in addition, supervised and reviewed the manuscript.

## Registration of research studies

NA.

## Guarantor

Dr. Eva Intagliata.

## Provenance and peer review

Not commissioned, externally peer-reviewed.

## Declaration of Competing Interest

There is no conflict of interest.
